# Supplementary material for: Structural analysis of S-ring composed of FliFG fusion proteins in marine Vibrio polar flagellar motor
Source: mBio. 2024 Sep 6;15(10):e01261-24. doi: 10.1128/mbio.01261-24 (PMC11481574; doi:10.1128/mbio.01261-24)
Supplement: Supplemental Figures and Tables — Fig. S1-S7 and Tables S1-S3. [file mbio.01261-24-s0001.pdf]

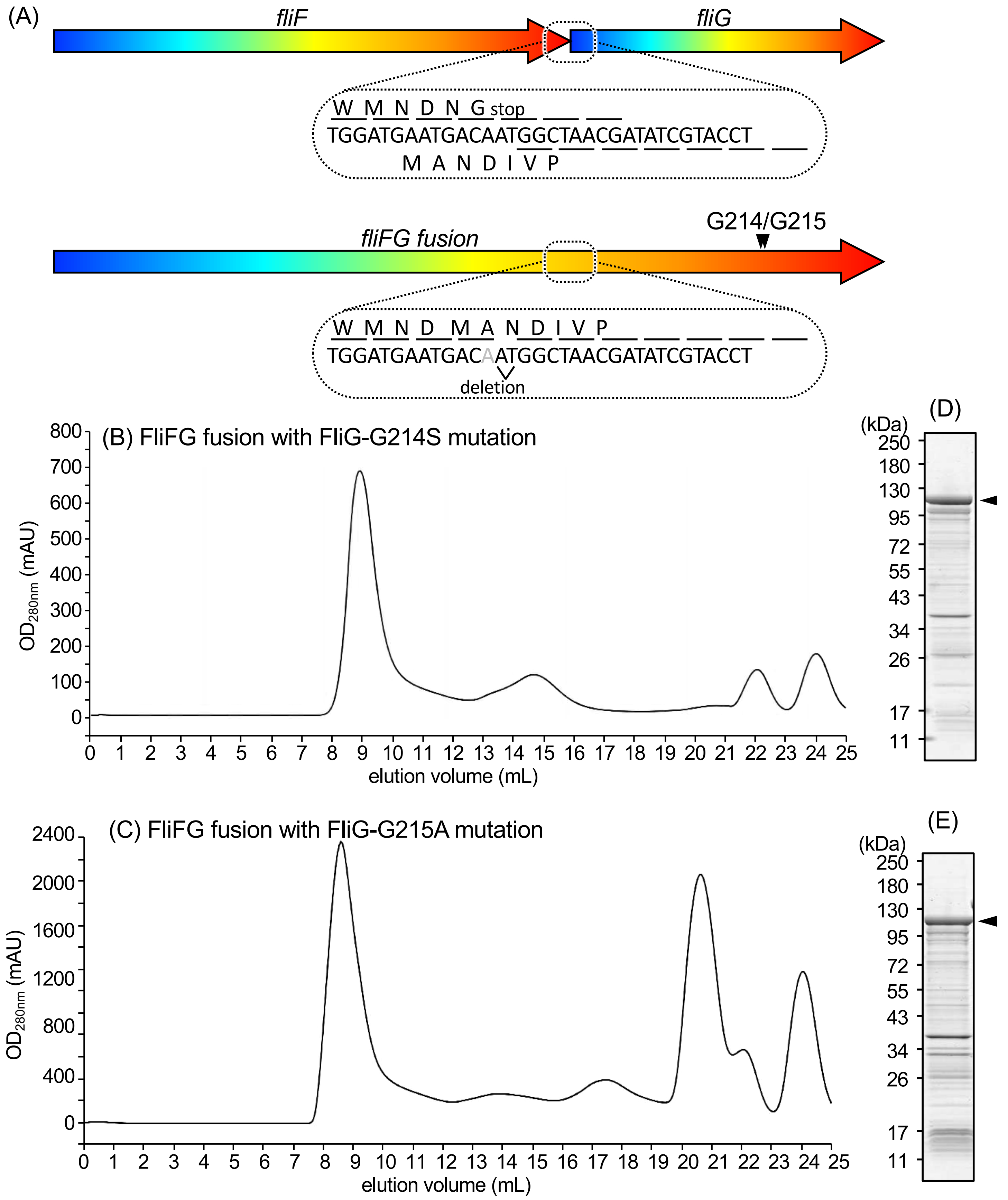

Fig. S1. Construction and purification of FliFG fusion proteins. (A) Schematic representation of *fliF*/*fliG* genes and the *fliFG* fusion gene of *Vibrio*. For the fusion, a single nucleotide just before start codon of the *fliG* gene was deleted, resulting in the loss of two C-terminal residues (Asn579 and Gly580) of FliF and fusion with the initial Met of FliG. (B and C) Elution profile of FliFG fusion proteins on the size exclusion column (Superose 6 10/300). The column was equilibrated with TK100L buffer, and 1.0 mL of FliFG fusior proteins with FliG-G214S mutation (B) or with FliG-G215A mutation (C) was loaded to the column and then eluted by TK100L buffer and 0.5 mL fractions were recovered. (D and E) SDS-PAGE profiles of the purified sample. The proteins in the fractions around elution volumes of 8–9 mL in (B) and (C) were separated by SDS-PAGE and stained with CBB for (D) and (E), respectively. Arrowheads indicate the position of FliFG fusion proteins.

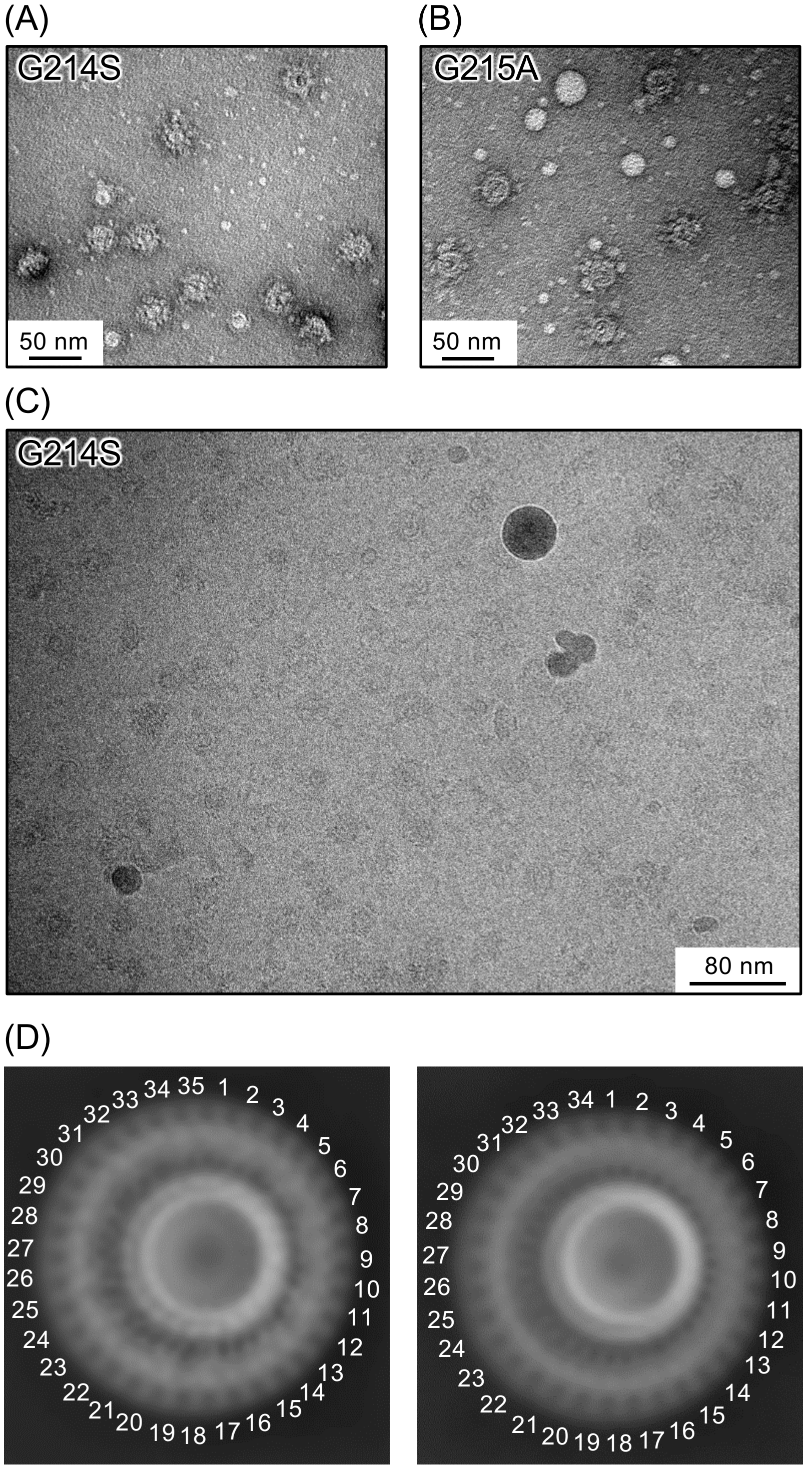

Fig. S2. Electron microscopy images of the MS-ring formed by FliFG fusion protein. (A and B) Negative staining images of the MS-ring with FliG-G214S mutation (A) or with FliG-G215A mutation (B). (C) Representative cryo-EM image of the MS-ring with FliG-G214S mutation. (D) The representative examples of the bottom views of S-ring with 35-fold (left) and 34-fold (right) symmetries in the 2D classification images.

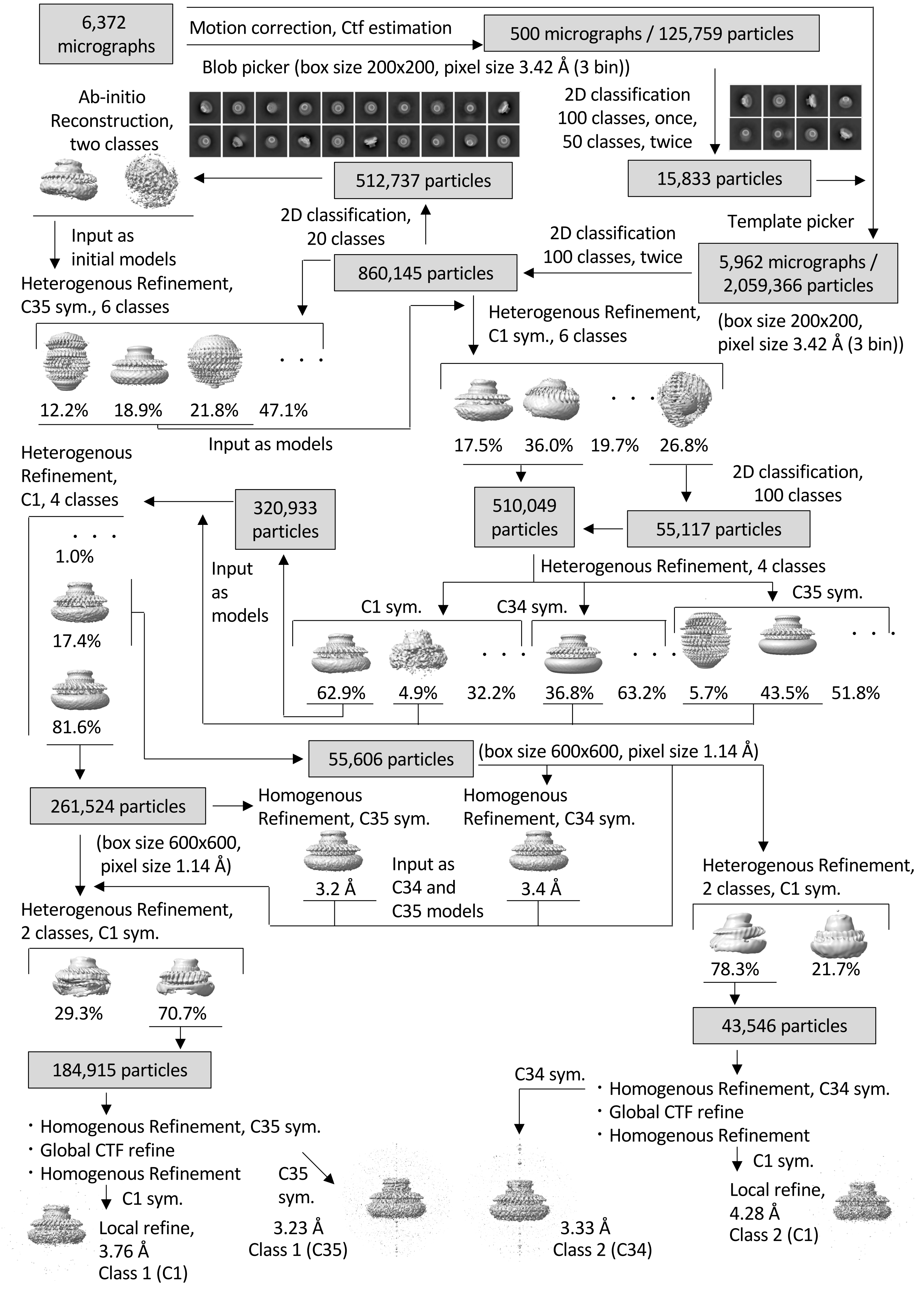

Fig. S3. Cryo-EM single particle 3D reconstruction scheme of the MS-ring formed by FliFG fusion protein with G214S mutation.

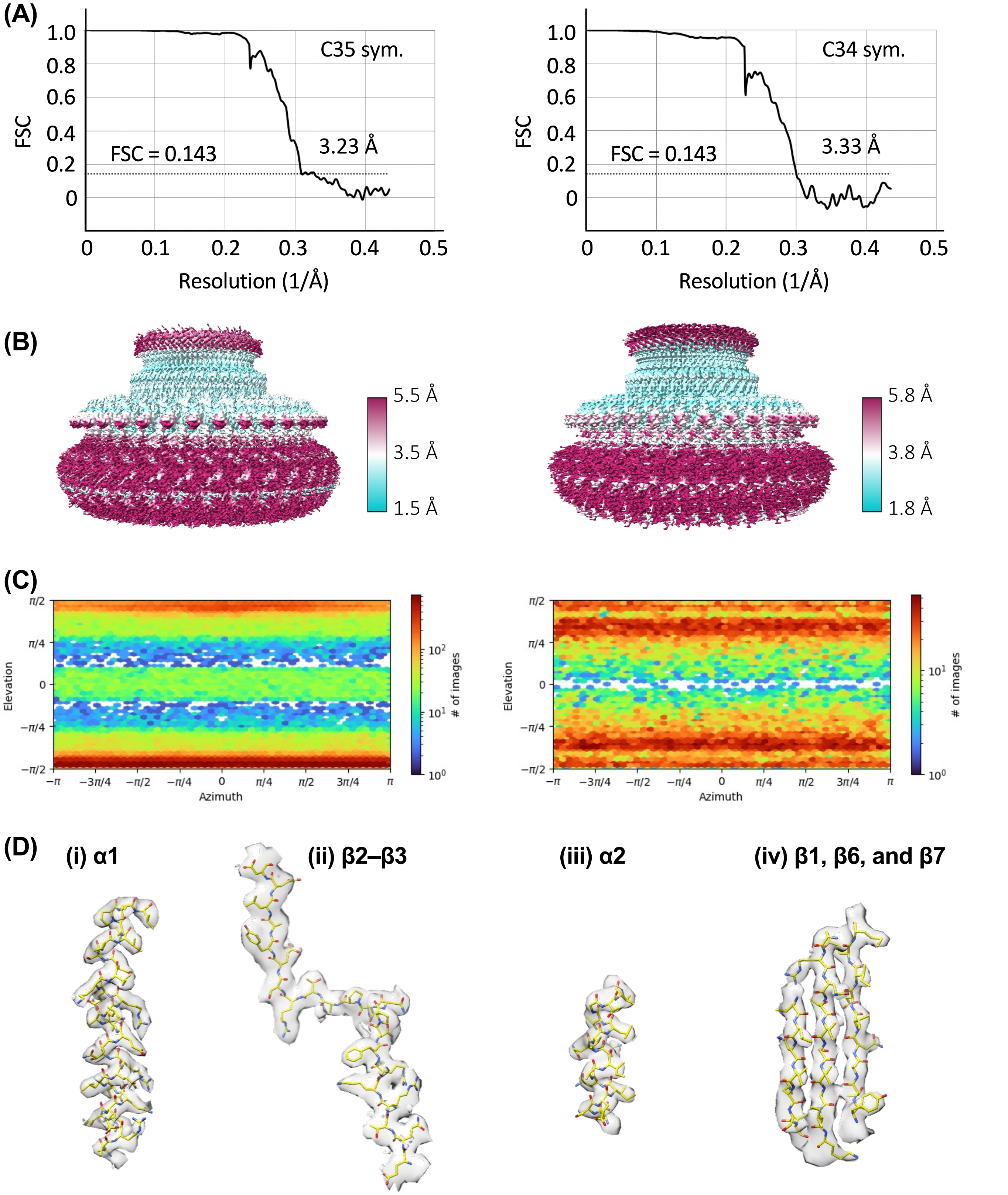

Fig. S4. Cryo-EM data analysis. (A) Fourier shell correlation curve of the map of Class 1 with C35 (left) and Class 2 with C34 (right). (B) Density maps colored by local resolution. (C) Direction distribution of particles used for 3D reconstruction. 3D volume with C35 and C34 symmetry are shown in the left and right panels, respectively. (D) Representative densities with their corresponding models in the map of Class 1 with C35 ((i) α1 and (ii) β2–β3) and Class 2 with C34 ((iii) α2 and (iv) β1, β6, and β7).

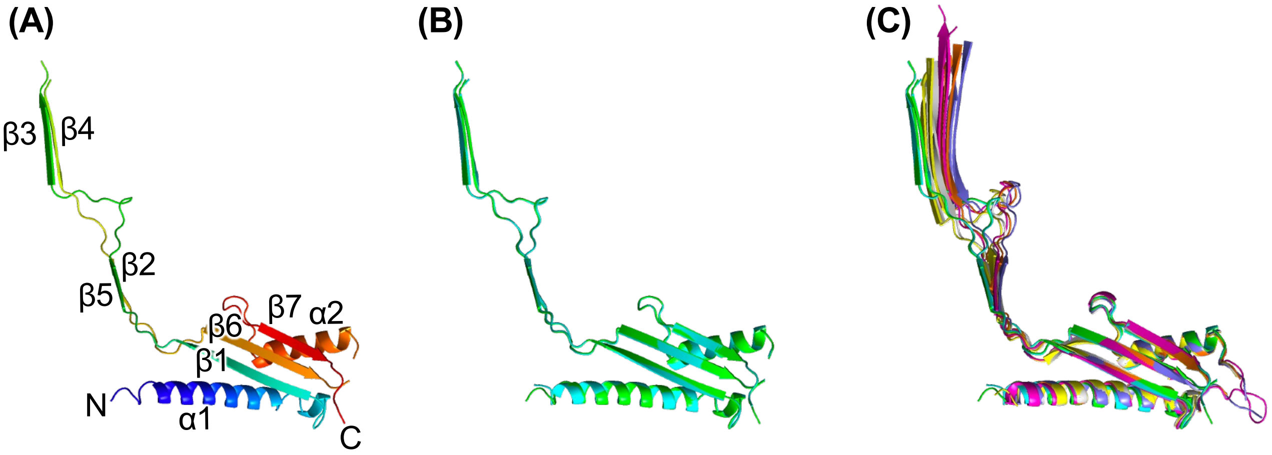

Fig. S5. Comparison of the various FliF protomer structures. (A) Magnified view of the protomer in the *Vibrio* S-ring of Class 1 (35-mer). (B) Superimposition of FliF protomer structures in the *Vibrio* S-rings, Class 1 (35-mer, green) and Class 2 (34-mer, cyan). (C) Superimposition of FliF protomer structures in the *Vibrio* and *Salmonella* S-rings. The superimposition was performed against the RBM3 region. Green: *Vibrio* S-ring (Class 1, 35-mer), cyan: *Vibrio* S-ring (Class 2, 34-mer), purple: PDB ID 7D84 (34-mer), magenta: 8FTF (33-mer), orange: 7CGO (34-mer), yellow: 6SCN (33-mer), gray: 6TRE (32-mer).

Takekawa et al. Fig. S5

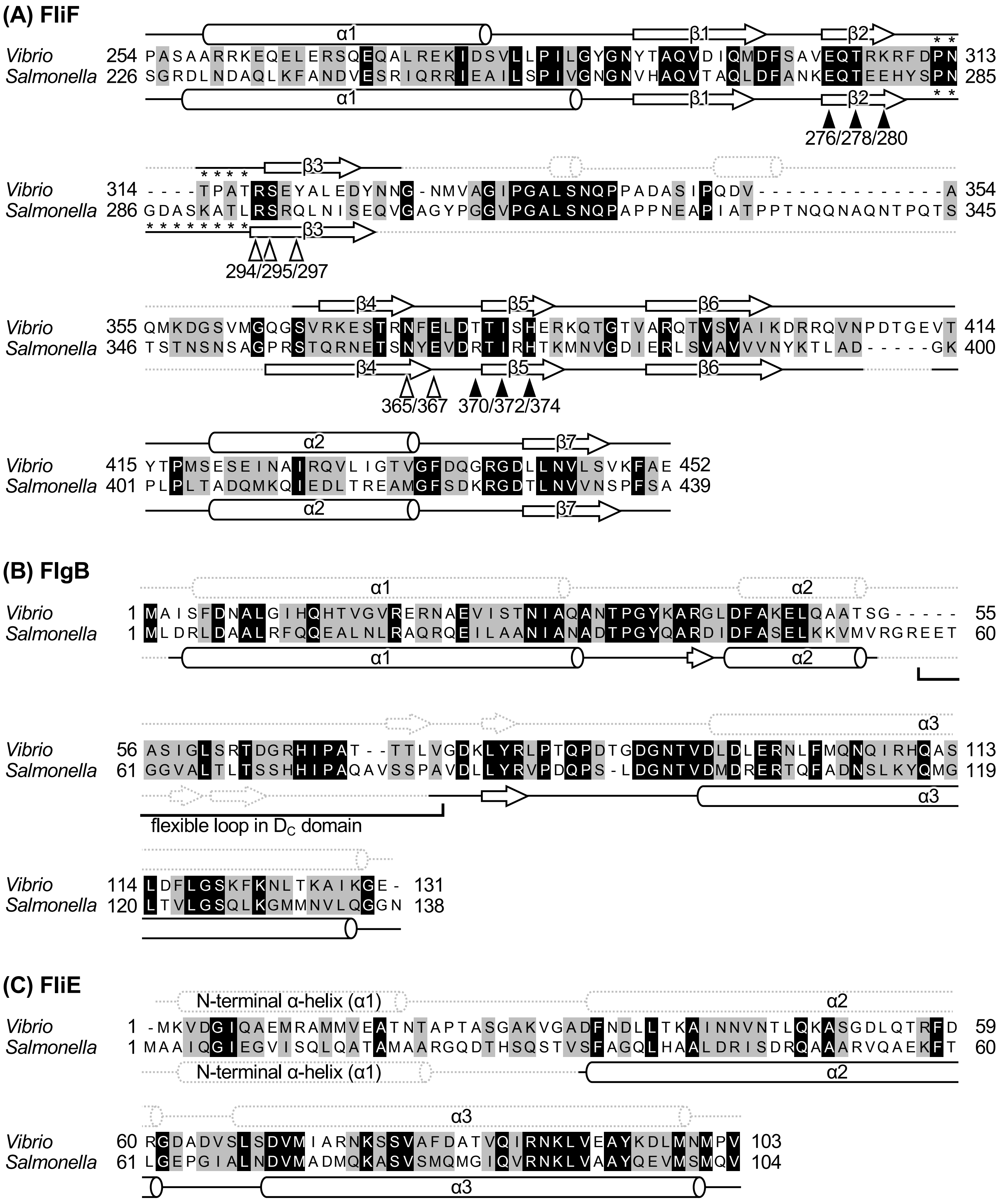

Fig. S6. Amino acid sequence alignment of RBM3–β-collar in FliF, FlgB and FliE from *Vibrio* and *Salmonella*. The identical and homologous amino acid residues are indicated in black and gray boxes, respectively. The secondary structures determined by the cryo-EM analysis and secondary structure prediction program (*PSIPRED*, http://bioinf.cs.ucl.ac.uk/psipred/) are shown by cylinders (α-helices) and arrows (β-strands). The residues interacting with FlgB and FliE in *Salmonella* FliF are shown by white and black arrow-heads, respectively. The flexible loop in the D$_C$ domain in FlgB and the N-terminal α-helix in FliE, which interact with the S-ring, are labeled.

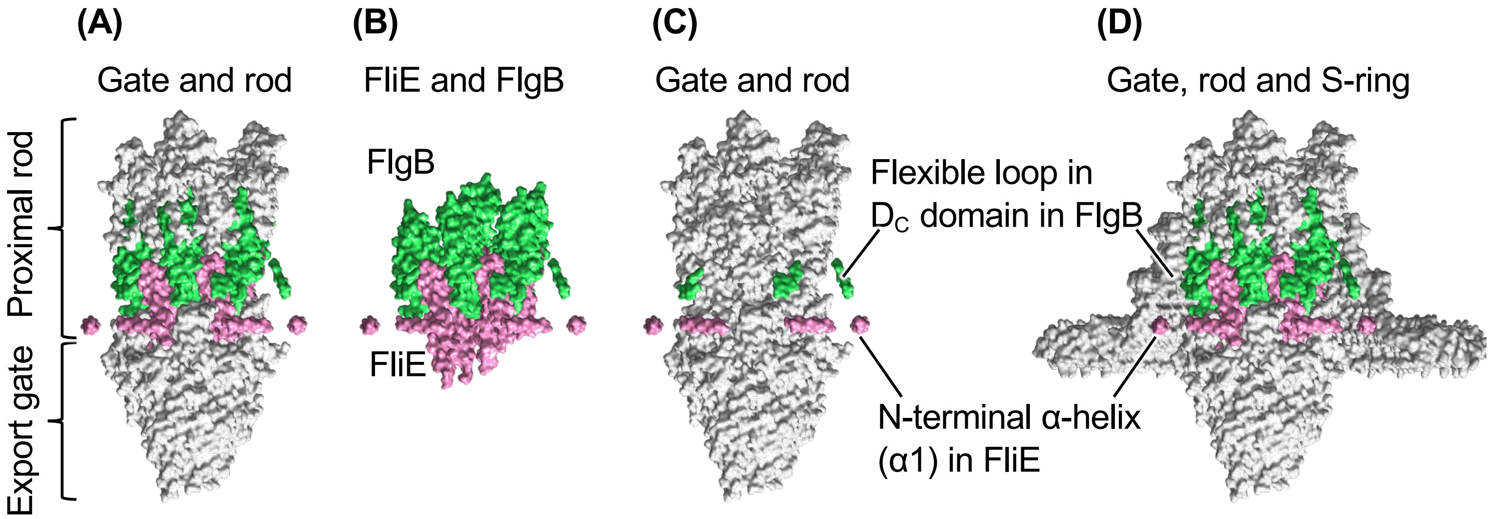

Fig. S7. Surface model of the previously reported export gate, proximal rod and S-ring structure (PDB ID: 7CGO). (A) Surface model of the export gate (FliP/FliQ/FliR) and the proximal rod (FliE/FlgB/FlgC) structures. Pink: FliE, green: FlgB. (B) Surface model of FliE and FlgB structures in the rod. (C) Surface model of the export gate and the proximal rod structures. Pink: N-terminal α-helix in FliE, green: D_C domain in FlgB. (D) Surface model of the export gate and the proximal rod structures with the cross-section image of the S-ring.

Legends for supplementary Movie

Movie S1 HS-AFM movies of the MS-ring composed of FliFG fusion proteins with FliG-G214S mutation. Real-time play speed: 0.15 s/frame.

Movie S2. HS-AFM movies of the MS-ring composed of FliFG fusion proteins with FliG-G214S mutation. Real-time play speed: 0.15 s/frame.

Table S1. Strains and plasmids used in this study.

Strains or plasmids	Genotype or description	Reference or source
E. coli strain		
DH5α	Recipient for DNA manipulation	(1)
BL21(DE3)	Host for protein expression	Novagen
Plasmids		
pCold I	Cold shock expression vector, Amp^r	Takara
pRO301	pCold I- *his-fliFG* fusion	(2)
pRO302	pCold I- *his-fliFG* fusion with FliG-G214S	This study
pRO303	pCold I- *his-fliFG* fusion with FliG-G215A	This study

Amp^r, ampicillin resistant.

(1) Grant SG, Jessee J, Bloom FR, Hanahan D. 1990. Differential plasmid rescue from transgenic mouse DNAs into Escherichia coli methylation-restriction mutants. Proc Natl Acad Sci USA 87:4645-4649.

(2) Takahashi K, Nishikino T, Kajino H, Kojima S, Uchihashi T, Homma M. 2023. Ring formation by *Vibrio* fusion protein composed of FliF and FliG, MS-ring and C-ring component of bacterial flagellar motor in membrane. Biophysics and Physicobiology 20:e200028.

Table S2. Summary of data correction and refinement statistics.

	Class 1 (C1)	Class 1 (C35)	Class 2 (C1)	Class 2 (C34)
Data collection and processing				
EM equipment	Taitan Krios	Taitan Krios	Taitan Krios	Taitan Krios
Detector	Gatan K3	Gatan K3	Gatan K3	Gatan K3
Energy filter	Gatan GIF Quantum, 20 eV slit	Gatan GIF Quantum, 20 eV slit	Gatan GIF Quantum, 20 eV slit	Gatan GIF Quantum, 20 eV slit
Magnification	64,000	64,000	64,000	64,000
Voltage (kV)	300	300	300	300
Electron exposure (e^-/ $Å^2$)	50	50	50	50
Defocus range (μm)	-0.7~-1.7	-0.7~-1.7	-0.7~-1.7	-0.7~-1.7
Pixel size (Å)	1.14	1.14	1.14	1.14
Symmetry imposed	C1	C35	C1	C34
Micrographs	6,372	6,372	6,372	6,372
Software	CryoSPARC	CryoSPARC	CryoSPARC	CryoSPARC
Initial particle images	2,059,366	2,059,366	2,059,366	2,059,366
Final particle images	184,915	184,915	43,546	43,546
Resolution (Å) / FSC threshold	3.76 / 0.143	3.23 / 0.143	4.28 / 0.143	3.33 / 0.143
**Refinement**				
Resolution (Å)	-	3.23	-	3.33
Model composition				
Non-hydrogen atoms	-	42,560	-	40,494
Protein residues	-	5,355	-	5,100
Ligands	-	0	-	0
RMSD				
Bonds length (Å)	-	0.003	-	0.003
Bonds angle (°)	-	0.603	-	0.550
Validation				
MolProbity score	-	1.32	-	1.44
Clashscore	-	5.86	-	7.10
Poor rotamers (%)	-	0.75	-	0.00
Ramachandran plot statistics (%)				
Preferred	-	98.64	-	97.79
Allowed	-	1.36	-	2.21
Outlier	-	0.00	-	0.00

Table S3. RMSD values of Cα atoms for superposed RBM3 structures. Values are in Å.

	RMSD against RBM3 of *Vibrio* FliF (34mer)
RBM3 of *Salmonella* FliF (6TRE)	1.260
RBM3 of *Salmonella* FliF (6SCN)	1.290
RBM3 of *Salmonella* FliF (8FTF)	1.110
RBM3 of *Salmonella* FliF (6SD3)	1.250
RBM3 of *Salmonella* FliF (7CGO)	1.155
RBM3 of *Salmonella* FliF (7D84)	1.124
RBM3 of *Vibrio* FliF (35mer)	0.251
